# Prenatal efavirenz exposure is independently associated with maternal, but not fetal *CYP2B6* genotype

**DOI:** 10.1097/FPC.0000000000000542

**Published:** 2024-06-24

**Authors:** Oluwasegun Eniayewu, Abdulafeez Akinloye, Babajide Shenkoya, Uche Azuka, Oluseye Bolaji, Ebunoluwa Adejuyigbe, Andrew Owen, Adeniyi Olagunju

**Affiliations:** a Department of Pharmaceutical Chemistry, Obafemi Awolowo University, Ile-Ife, Nigeria; b Department of Pharmaceutical and Medicinal Chemistry, University of Ilorin, Ilorin, Nigeria; c Department of Obstetrics and Gynaecology, Federal Medical Centre, Makurdi, Nigeria; d Department of Paediatrics and Child Health, Obafemi Awolowo University, Ile-Ife, Nigeria; e Department of Pharmacology and Therapeutics, University of Liverpool, Liverpool, United Kingdom

**Keywords:** pregnancy, pharmacogenetics, prenatal, efavirenz, *CYB2B6*

## Abstract

**Objectives:**

Understanding the influence of fetal and maternal genetics on prenatal drug exposure could potentially improve benefit-risk evaluation. In this study, we investigated the impact of two functional polymorphisms in *CYP2B6* on prenatal exposure to efavirenz.

**Methods:**

Dried blood spot (DBS) samples were collected from HIV-positive pregnant women (*n* = 112) and their newborns (*n* = 107) at delivery. They were genotyped for single nucleotide polymorphisms in *CYP2B6*. Efavirenz was quantified by liquid chromatography-tandem mass spectrometry (LC-MS/MS).

**Results:**

Significant correlations were observed in efavirenz concentration between maternal and newborn (*r* = 0.46, *R*^2^ = 0.21, *P* < 0.001), and maternal and cord (*r* = 0.83, *R*^2^ = 0.68, *P* < 0.001) samples. Median (interquartile range) newborn plasma-to-maternal plasma and cord-to-maternal plasma ratios were 0.85 (0.03–3.49) and 0.78 (0.23–1.96), respectively. Newborn efavirenz concentration in DBS varied significantly based on composite maternal *CYP2B6* genotype: fast (*CYP2B6* 516GG and 983TT, *n* = 26), 747 ng/ml (602–1060); intermediate (*CYP2B6* 516GT or 983TC *n* = 50), 1177 ng/ml (898–1765); and slow (*CYP2B6* 516GT and 983TC or 516TT or 983CC, *n* = 14), 3094 ng/ml (2126–3812). Composite newborn *CYP2B6* genotype was, however, not significantly associated with prenatal exposure. Efavirenz concentration in newborn stratified as fast (*n* = 25), intermediate (*n* = 36), and slow metabolizers (*n* = 19) from prenatal exposure was 999.7 (774–1285), 1240 (709–1984), and 1792 ng/ml (1201–3188).

**Conclusions:**

The clinical relevance of the observed influence of maternal genetics on prenatal efavirenz exposure requires further investigation.

## 1.0 Introduction

Prescription medicines are frequently prescribed during pregnancy to manage different medical conditions [1,2]. These medications may potentially cross the placental barrier, and thus expose the fetus to varying levels of maternal drug exposures at different stages of pregnancy [3,4]. Pharmacogenetics plays a key role in explaining between-subject variability of drug exposures in pregnant adults [5]. Additionally, the possibility that a fetus may express a different polymorphism for a drug-metabolizing enzyme than its mother adds further uncertainty to subsequent fetal drug exposure.

Fetal exposure to drugs administered to the mother can result in several adverse clinical outcomes, including fetal malformation and death [6]. Therefore, understanding the impact of polymorphism in drug metabolizing enzyme genes on fetal drug disposition is crucial in optimizing drug dosing during pregnancy and mitigating harm to the fetus. However, due to the invasive nature of direct fetal sampling procedures, our understanding of fetal exposure to most drugs and associated factors influencing drug exposure is limited. Studies that investigate prenatal drug exposure to maternal drugs are critical in addressing this gap in knowledge.

Efavirenz is a non-nucleoside reverse transcriptase inhibitor that blocks the transcription of HIV replication [7–10]. It is primarily metabolized by the highly polymorphic hepatic *CYP2B6* enzymes [11–14]. The drug exhibits differential pharmacokinetics among individuals due to factors such as pregnancy, ethnicity, weight, *CYP2B6* variant, and drug-drug interactions [15–18]. Notably, single nucleotide polymorphisms (SNPs) in the *CYP2B6* gene at positions 516 (*CYP2B6* 516G>T, rs3745274), 983 (*CYP2B6* 983T>C, rs28399499), and 15582 (*CYP2B6* 15582 T>C, rs4803419) are the significant predictors of efavirenz exposure [19–22], with *CYP2B6* 516G>T rs3745274) and 983T>C (rs28399499) being more prevalent in individuals of African ancestry [23–25].

Although pregnancy and *CYP2B6* polymorphism have been established to alter plasma drug exposure [26,27], their impact on fetal exposure to maternal drugs remains poorly understood. In this study, we performed a comprehensive maternal-newborn genetic analysis and determined the impact of two functional polymorphisms in *the CYP2B6* gene on newborn exposure to efavirenz.

## 2.0 Method

### 2.1 Study participants and design

Participants in this study were HIV-positive pregnant women recruited into the VADICT study (ClinicalTrials.gov Identifier: NCT03284645) and receiving an efavirenz-based Antiretroviral Therapy (ART) regimen. Participants in the VADICT study were recruited at the Federal Medical Centre and the Bishop Murray Medical Centre, both in Benue State, Nigeria. Study inclusion criteria include pregnant women receiving efavirenz-based ART, planned exclusive breastfeeding until 6 months post-delivery, and the ability of participants to understand study information and comply with the sampling procedure and follow-up schedule. Participants were excluded from the study if they had any serious illness or were taking drugs or herbal medications that could potentially interact with efavirenz. A total of 103 mother-newborn paired dried blood spot (DBS) samples were collected from 112 pregnant women and 107 newborns at delivery. Approval of the study protocol and material transfer agreements were obtained from the National Health Research and Ethics Committee of Nigeria (NHREC/01/01/2007-05/06/2017) and the Research Ethics Committees of the participating hospitals.

### 2.2 Sampling

Maternal and newborn DBS samples were collected at a single recorded time-point post dose by cord clamping, heel pricking of the newborn, or maternal finger pricking at the time of delivery (before the start of breastfeeding), and accurately spotting blood drops on Whatman protein saver cards (GE Healthcare, Little Chalfont, Buckinghamshire, UK). All samples were dried at room temperature in open air for 3-4 h, packed in ziplock bags (with desiccants) for storage at -80°C until the time of analysis. In addition to the blood samples, maternal and newborn-associated variables such as time post-dose, body weight, BMI, gestational age, gravidity, and APGAR (Appearance, Pulse, Grimace, Activity and Respiration) were also collected.

### 2.3 Pharmacokinetic analysis

The concentration of efavirenz in maternal and newborn DBS samples was analyzed at the Translational Pharmacokinetic Laboratory, Faculty of Pharmacy, Obafemi Awolowo University, Nigeria, using a liquid chromatography-tandem mass spectrometry (LC-MS/MS) method. The plasma concentrations of efavirenz was extrapolated from maternal and newborn DBS concentrations, hematocrit level in women and fraction of efavirenz bound to plasma proteins using a previously described formula [28,29].

### 2.4 Pharmacogenetic analysis

Two *CYP2B6* polymorphisms (*CYP2B6* 516G>T; rs3745274; and *CYP2B6* 983T>C; rs28399499) known to affect efavirenz disposition and exposure were analyzed in the maternal and newborn DBS samples. E.Z.N.A. Blood DNA Mini Kits (Omega Bio-Tek, Norcross, Georgia) were used to extract the genomic DNA following the manufacturer’s specifications. The extracted DNA was quantified by measuring the amount of light absorbed using NanoDrop (Thermo Fisher Scientific, Wilmington, Delaware). Genotyping of polymorphisms of interest was carried out by a real-time polymerase chain reaction assay on a DNA Engine Chromo4 system (Bio-Rad Laboratories, Hercules, California): involving denaturation (95 °C, 15 minutes), amplification (95 °C, 50 cycles, 15 seconds), and annealing (60 °C, 1 minutes). Allelic discrimination plots from the TaqMan Genotyping Master Mix and assays on Opticon Monitor software v 3.1 (Bio-Rad Laboratories) were used for allele assortment.

Composite *CYP2B6* genotypes were grouped based on their impact on efavirenz exposure as fast (no variant allele at position 516 or 983), intermediate (variant allele at either position 516 or 983), or slow (variant allele at both positions 516 and 983).

### 2.5 Statistical analysis

Maternal- and newborn-associated variables collected were summarized using descriptive statistics. Pearson correlation was used to assess the correlation between maternal, newborn and cord efavirenz concentrations. Variables associated with efavirenz newborn concentration were determined using a univariate linear regression analysis. Independent variables with probability values lesser or equal to 0.05 were included in a multivariate linear regression analysis The difference in the mean concentrations stratified by genotype was also evaluated using Mann-Whiney and Kruskal Wallis tests. All statistical analyses were executed using the IBM SPSS Statistics v 21.0.0.0 (IBM, Armonk, New York) software and all charts were plotted using the GraphPad Prism 5 (GraphPad Software Inc., La Jolla, California).

## 3.0 Results

### 3.1 Participants

A total of 277 samples (105 maternal, 107 newborn and 65 cord samples) were obtained from 112 pregnant women and 107 of their newborns at delivery. Paired maternal-newborn (n = 103) and newborn-cord (n = 62) samples were analyzed. The median (IQR) age and body weight of the 112 pregnant women was 30.7 years (26.5-34.2) and 71.0 kg (63.0-83.0), respectively. Maternal samples were collected at delivery (mean gestational age of 39.7 weeks) 17.4 h after the last dose.

Based on their *CYP2B6* metabolizer status, study participants (mothers and newborn) were stratified into fast, intermediate, and slow metabolizers of efavirenz. Details of the baseline demographic information and genotype frequencies of both the mother and the newborn is presented in Table 1.

### 3.2 Maternal and newborn efavirenz pharmacokinetics

**Figure 1:**
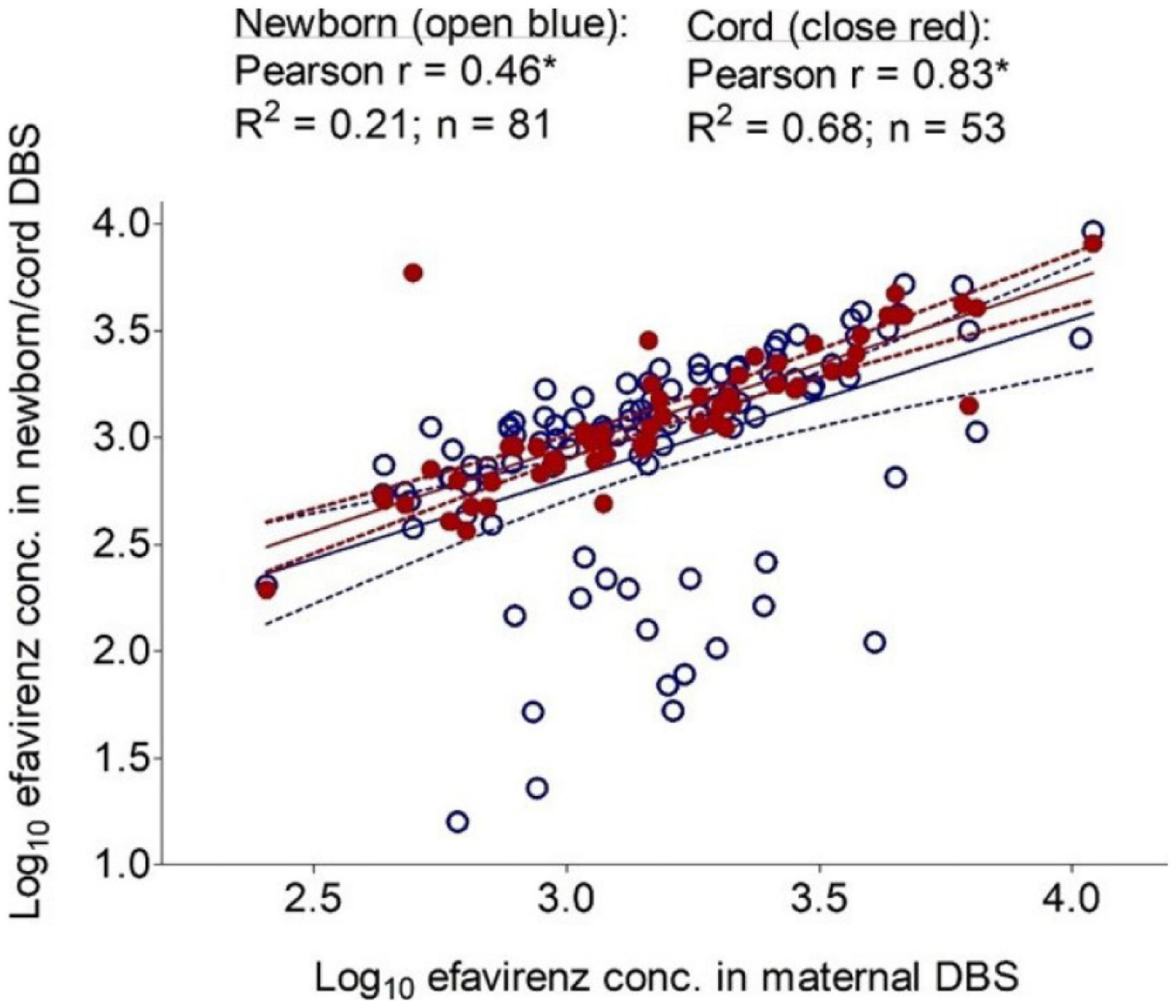
Correlation between efavirenz maternal and newborn DBS concentrations (open blue circle), Pearson’s r = 0.46; and maternal and cord DBS concentrations (solid red circles), Pearson’s r = 0.83. Solid lines represent mean lines and 95% confidence interval.

The median (IQR) efavirenz DBS concentration in maternal (n = 105) and newborn (n = 107) DBS samples at delivery 17.4 h after the last dose were 1420 ng/mL (792-2358) and 1118 ng/mL (576-1803), respectively. The corresponding plasma concentrations were 2047 ng/mL (1141-3470) and 2023 ng/mL (1003-3284), respectively. Efavirenz cord concentration estimated from 65 cord samples was 1074 ng/ml (736-1957). There was a positive correlation between efavirenz maternal and newborn concentration (Pearson’s r = 0.46) and efavirenz maternal and cord concentration ((Pearson’s r = 0.83)(Figure 1). Median (IQR) newborn plasma -to-maternal plasma and cord-to-maternal plasma ratios was estimated to be 0.85 (0.03-3.49) and 0.78 (0.23-1.96), respectively.

### 3.3 Impact of maternal and newborn *CYP2B6* genotype on efavirenz newborn exposure

**Figure 2:**
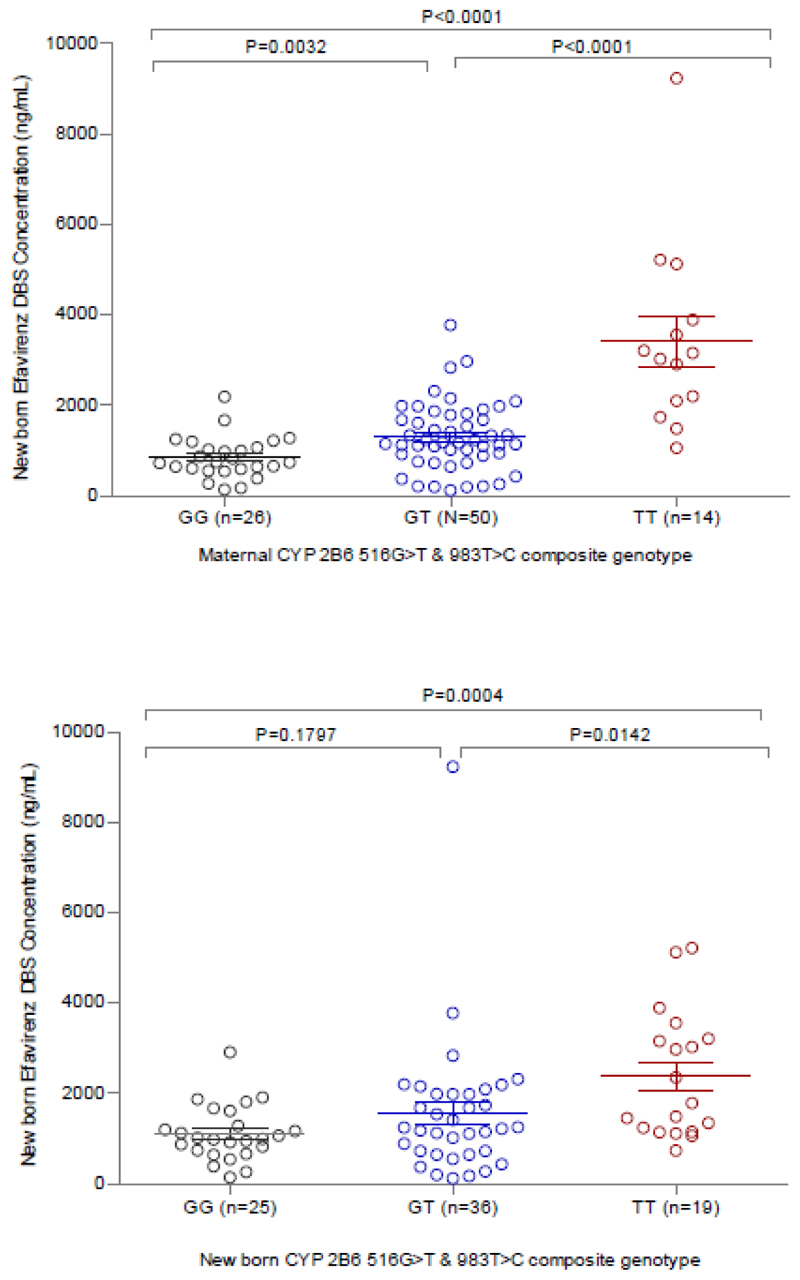
Associations between composite maternal *CYP2B6* (fast, n = 26; intermediate, n = 50; slow, n = 14) and newborn efavirenz DBS concentrations (A); and composite newborn *CYP2B6* (fast, n = 25; intermediate, n = 36; slow, n = 19) and newborn efavirenz DBS concentrations (B). Bars represent mean (SEM), and *P* values are for Kruskal-Wallis test.

The result of the univariate analysis indicates that maternal age at delivery (*P =* 0.955), gestational age (*P* = 0.422), and time after maternal dose (*P* = 0.141) were not significantly associated with newborn efavirenz concentrations. On the other hand, both maternal (*P* = 2.89 x 10^-4^) and newborn (*P* = 6.47 x 10^-4^) *CYP2B6* genotypes, APGAR score (*P* = 0.0014) and newborn BMI (*P* = 0.028) were associated with efavirenz newborn DBS concentration at 95% confidence interval. On multivariate analysis, only maternal *CYP2B6* genotype and APGAR score remained significantly (*P* = 6.3 x 10^-4^) associated with newborn efavirenz concentration (Table 2). After correcting for efavirenz maternal plasma concentration, we observed that the association between maternal *CYP2B6* and newborn efavirenz concentration remained significant (p= 0.013) although the percentage variance in the newborn efavirenz concentration that can be explained by maternal *CYP2B6* (indicated by R square changes) reduced from 13.2% to 5.1%. These findings suggest that newborn *CYP2B6* genotypes is not a strong predictor of newborn efavirenz concentration. The association between *CYP2B6* genotype (maternal and newborn) and efavirenz newborn concentration is presented in Figure 2.

Efavirenz newborn concentration varies based on both *CYP2B6* 516 G>T (rs3745274) and *CYP2B6* 983 T>C (rs28399499) newborn genotype (Table 3). The median (IQR) efavirenz DBS concentration in newborn stratified as fast (*CYP2B6* 516 GG, n = 25, intermediate (*CYP2B6* 516 GT, n = 36), and slow metabolizers (*CYP2B6* 516 TT, n = 19) 17.4 h after maternal dose were 999.7 ng/ml (744-1285), 1240 ng/ml (709-1984) and 1792 ng/ml (1201-3188), respectively. Similarly, newborn efavirenz DBS concentration varied based on maternal *CYP2B6* genotypes: fast metabolizers (n = 26), 747 ng/ml (602-1060); Intermediate metabolizers (n = 50), 1177 (898-1765); and slow metabolizers (n = 14), 3094 (2126 3812). Additionally, we observed a genotype effect on efavirenz concentrations in newborns when stratified by *CYP2B6* genotype within each maternal reference group (fast, intermediate, and slow). Newborns with a slow metabolizer status, born to mothers with intermediate or slow metabolizer statuses, had higher efavirenz plasma exposure. We could not establish this trend for slow metabolizer newborns from fast metabolizer mothers, as none of the 22 mother-newborn pairs in this category included a slow metabolizer newborn. (Table 4)

## 4.0 Discussion

Genetics is a key factor for interindividual variability in efavirenz disposition within a population. Polymorphism in the gene affecting the functionality of hepatic enzymes often has a significant impact on drug exposure. Despite extensive studies investigating the relationship between *CYP2B6* polymorphisms and efavirenz exposure [24,25,27,30], the pharmacokinetics and pharmacogenetics in prenatal efavirenz exposure has not been fully understood. In this study, we reported the pharmacokinetics of efavirenz in maternal-newborn pair stratified based on their composite *CYP2B6* genotype at delivery.

The use of antiretroviral drugs in pregnancy is beneficial to both the mother and the fetus [31,32]. Despite the pharmacokinetic alteration of drugs due to pregnancy-induced physiological changes [33], fetal safety limits drug studies in pregnancy. These uncertainties may be responsible for the poor understanding of the exposure of the fetus to medication used during pregnancy. In this study, a DBS technique was used to measure efavirenz concentration in mothers and newborns because it is a convenient and non-invasive method that requires minimal blood volume, can be easily stored and shipped, and allows for simplified sampling [34]. DBS-derived plasma concentrations were calculated using: [concentration of efavirenz in DBS / (1 – haematocrit)] x fraction of efavirenz bound to plasma proteins [29]. Consistent with previously reported [28,29,35], the DBS efavirenz level measured in this study was 31% lower than the calculated theoretical plasma concentration. Nevertheless, the efavirenz DBS concentrations were used in the correlation analyses. The results of this study showed a strong correlation between efavirenz concentration in newborn and maternal plasma and cord blood, suggesting that maternal efavirenz concentration is a potent predictor of fetal concentration. In previous studies, higher efavirenz exposure has been shown to be associated with weight loss [15,36,37], probably due to a lower volume of distribution and clearance.

Several studies have reported an increase in efavirenz concentration due to polymorphisms in *CYP2B6* genotype. Specifically, most of these studies showed that individuals with *CYP2B6* 516 TT genotype experienced up a to three-fold increase in efavirenz exposure [38–40]. In our study, we observed that maternal *CYP2B6* 516G>T was significantly (p = 2.0 x 10^-8^) associated with maternal efavirenz concentration, with individuals with the *CYP2B6* 516 TT genotype having close to 3.8 fold increase in maternal efavirenz concentration compared to those with the *CYP2B6* 516 GG genotype. Although the association between the maternal *CYP2B6* 983T>C and efavirenz newborn concentration was not significant (*P* = 0.174), our study showed a significant association between maternal *CYP2B6* 516G>T and efavirenz newborn concentration (*P* = 0.0018). The absence of significant association between maternal *CYP2B6* 983T>C genotype and efavirenz newborn concentration may be due the skewness in the *CYP2B6* 983T>C (rs28399499) genotype data used in our study, with TT and TC constituting 89% and 11% of the dataset, respectively. In the pooled analysis, composite maternal *CYP2B6* genotype (*CYP2B6* 516G>T and *CYP2B6* 983T>C) was significantly associated with efavirenz newborn concentration before and after adjusting for maternal efavirenz concentration (Table 2).

Kruskal-Wallis’s test showed that there is a significant difference between the median efavirenz concentration among newborns with fast, intermediate, and slow metabolizer status. Consistent with previous studies that polymorphisms in *CYP2B6* 516G>T (rs3745274) and 983T>C (rs28399499) are associated with elevated plasma concentration of efavirenz [17,30,41–44], we showed that *CYP2B6* 516TT and 983CC SNPs resulted in higher efavirenz newborn concentrations. Higher concentrations of efavirenz were observed in newborns with slow metabolizer status, predominantly when their mothers exhibited intermediate or slow metabolizer statuses. This phenomenon likely arises from the immaturity of cytochrome P450 enzymes in newborns, potentially predisposing them to drug-related toxicity from maternal drug dosing. Therefore, identifying a newborns’ metabolizer status is critical for mitigating the risk of fetal exposure to maternal antiretroviral drugs during pregnancy.

The univariate regression showed that the APGAR score, the newborn’s BMI and *CYP2B6* genotype, and the mother’s *CYP2B6* genotype were significant predictors of efavirenz concentrations in the newborn. Based on our findings, low neonatal APGAR scores and a high BMI are likely associated with high efavirenz exposure. While there has not been an established link between APGAR score and efavirenz concentration in neonates, a gentamicin study in neonates identified APGAR score as a strong predictor of drug disposition [45]. A high APGAR score indicates a newborn’s good health status, and the negative correlation of newborn efavirenz concentration with the APGAR score suggests that there is a serious concern for fetal health with increasing efavirenz concentration. In the multivariate regression step to adjust for possible confounding variables, we observed that the newborn *CYP2B6* genotype is no longer a significant predictor of newborn efavirenz concentration, but maternal *CYP2B6* genotype and APGAR score remained significant. This may largely be a result of immature CYP enzymes in the fetus/newborn; hence the fetus relies on the mother’s CYP enzymes for metabolism during pregnancy [46,47].

Understanding the impact of polymorphisms in drug disposition genes on fetal drug exposure is crucial in optimizing drug safety during pregnancy. This is especially important for drugs with significant genetic contribution to variability. Though efavirenz was used in this study, the findings reported here could have a broader translational potential, including in-silico modelling of fetal exposure to maternal drugs.

The current study showed that the maternal *CYP2B6* genotype independently influences prenatal exposure to efavirenz. Knowledge of maternal pharmacogenetics may help rationalize drug selection by informing the risk-benefit ratio during pregnancy.

## Figures and Tables

**Table 1:** Characteristics of mothers and newborn at delivery.

Characteristics^a^	Mother at delivery	Newborn
Maternal age (years)	31 (27-34)	NA
Maternal weight (kg)	71 (63-83)	NA
Gravidity	2 (2-4)	NA
Post-dose sampling time (h)	15 (12-23)	16 (12-23)
APGAR score	NA	8 (8-9)
Baby birth weight (kg)	NA	3.0 (2.7-3.2)
Baby body mass index (kg/m^2^)	NA	6.1 (5.6-7.1)
**Genotype frequencies (%)**		
***CYP2B6* 516 G>T (rs3745274)**	(n = 104)	(n = 85)
GG	39.4	42.4
GT	46.2	38.8
TT	14.4	18.8
***CYP2B6* 983 T>C (rs28399499)**	(n = 105)	(n = 85)
TT	89.5	89.4
TC	10.5	9.40
CC	0.00	1.20
***CYP2B6* metabolizer phenotype^b^**	(n = 102)	(n = 86)
Fast metabolizer	30.4	33.7
Intermediate metabolizer	52.9	44.2
Slow metabolizer	16.7	22.1

APGAR, appearance, pulse, grimace, activity and respiration.

a Data presented in median (interquartile range).

b Based on composite *CYP2B6* 516G>T and 983T>C genotypes: fast metabolizers, participants with no variant allele at both positions; intermediate metabolizers, variant allele at either position; slow metabolizers, one variant allele each at both positions or two variant alleles at either position.

**Table 2:** Univariate and multivariate regression analyses for association of genetic and non-genetic variables with maternal and newborn efavirenz concentration.

	Univariate linear regression		Multivariate linear regression	
Patient characteristics	β^a^ (log_10_ Efavirenz conc., 95% CI)	*P value*	β (log_10_ EFV conc., 95% CI)	*P value*
**Maternal age at** **delivery (years)**				
Maternal plasma	0.01 (-0.005, 0.03)	0.173		
Newborn plasma	0.001 (-0.02, 0.02)	0.955		
**Gestational age** **(weeks)**				
Maternal plasma	0.002 (-0.022, 0.026)	0.857		
Newborn plasma	-0.01 (-0.05, 0.02)	0.422		
**Maternal body weight** **(kg)**				
Maternal plasma	-0.003 (-0.011, 0.005)	0.423		
Newborn plasma	0.001 (-0.011, 0.013)	0.837		
**Newborn body weight** **(kg)**				
Maternal plasma	-0.05 (-0.22, 0.12)	0.539		
Newborn plasma	0.09 (-0.15, 0.35)	0.439		
**Newborn BMI (kg/m^2^)**				
Maternal plasma	0.02 (-0.01, 0.06)	0.180		
Newborn plasma	0.06 (0.01, 1.11)	0.027	Excluded	-
**Time after maternal** **dose**				
Maternal plasma	-0.005 (-0.013, 0.003)	0.249		
Newborn plasma	-0.02 (-0.03, 0.01)	0.141		
**APGAR score**				
Newborn plasma	-0.15 (-0.24, -0.06)	1.4 x 10^-3^	-0.15 (-0.23, -0.07)	6.0 x 10^-4^
**Maternal *CYP2B6*** **516G>T (rs3745274)**				
Maternal plasma	0.30 (0.20, 0.40)	4.1 x 10^-8^	0.24 (0.08, 0.40)	2.0 x 10^-8^
Newborn plasma	0.24 (0.09, 0.40)	2.6 x 10^-3^	0.25 (0.10, 0.41)^b^	1.8 x 10^-3^
**Newborn *CYP2B6*** **516G>T (rs3745274)**				
Maternal plasma	0.12 (0.03, 0.22)	0.011	Excluded	-
Newborn plasma	0.24 (0.12, 0.37)	2.8 x 10^-4^	Excluded	-
**Maternal *CYP2B6*** **983T>C (rs28399499)**				
Maternal plasma	0.12 (-0.14, 0.38)	0.378		
Newborn plasma	0.26 (-0.12, 0.63	0.174		
**Newborn *CYP2B6*** **983T>C (rs28399499)**				
Maternal plasma	0.01 (-0.19, 0.21)	0.921		
Newborn plasma	-0.02 (-0.31, 0.27)	0.877		
**Maternal composite** ***CYP2B6* genotype**				
Maternal plasma	0.31 (0.23, 0.40)	2.4 x 10^-9^	0.30 (0.21, 0.39)	3.7 x 10^-9^
Newborn plasma	0.27 (0.13, 0.42)	2.9 x 10^-4^	0.30 (0.13, 0.47)	6.3 x 10^-4^
**Newborn composite** ***CYP2B6* genotype**				
Maternal plasma	0.13 (0.03, 0.22)	0.009	Excluded	-
Newborn plasma	0.24 (0.11, 0.37)	6.47 x 10^-4^	Excluded	-

APGAR, appearance, pulse, grimace, activity and respiration; BMI, Body mass index; Cl, confidence interval.

a β, regression coefficient which represents incremental change in log_10_ efavirenz concentration per unit change in a patient characteristic.

b The association between maternal *CYP2B6* genotype and efavirenz newborn concentration remained significant (*P value* = 0.013) after correcting for maternal plasma efavirenz concentration.

**Table 3:** Median (IQR) efavirenz concentration grouped by maternal and newborn *CYP2B6* genotype.

	Newborn	Mother
**Maternal *CYP2B6* genotype**		
*CYP2B6* 516 G>T (rs3745274)		
GG (n = 37/38)^a^	749 (391-1226)	996 (702-1459)
GT (n = 48/47)	1150 (734-1681)	1444 (945-2113)
TT (n = 15/15)	2914 (1277-3389)	3818 (2977-5360)
*P* value^b^	0.001	< 0.001
*CYP2B6* 983 T>C (rs28399499)		
TT (n = 88/88)	1432 (885-2382)	1115 (649-1681)
TC (n = 11/10)	1712 (1012-2392)	1948 (1081-2099)
CC	-	-
*P* value	0.084	0.462
Maternal *CYP2B6* metabolizer phenotype		
Fast metabolizers (n = 26/30)	747 (602-1060)	867 (657-1294)
Intermediate metabolizers (n = 50/54)	1177 (898-1765)	1432 (916-2053)
Slow metabolizers (n = 14/17)	3094 (2126-3812)	3818 (2870-6070)
*P* value	< 0.001	< 0.001
**Newborn *CYP2B6* genotype**		
*CYP2B6* 516 G>T (rs3745274)		
GG (n = 32)	1012 (680-1367)	
GT (n = 33)	1253 (730-1982)	
TT (n = 16)	2073 (1299-3174)	
*P* value	< 0.001	
*CYP2B6* 983 T>C (rs28399499)		
TT (n = 73)	1206 (828-1915)	
TC (n = 8)	1091 (583-2281)	
CC (n = 1)	1246	
*P* value	0.965	
Newborn *CYP2B6* metabolizer phenotype		
Fast metabolizers (n = 25)	999.7 (744-1285)	
Intermediate metabolizers (n = 36)	1240 (709-1984)	
Slow metabolizers (n = 19)	1792 (1201-3188)	
*P* value	0.001	

IQR, interquartile range.

a n = 37/38, 37 newborns and 38 mothers.

b *P-*values were derived from either Kruskal–Wallis (for comparison between three groups), or Mann–Whitney (for comparison between two groups) test statistics.

**Table 4:** Efavirenz concentration in mothers and newborns grouped by maternal and newborn *CYP2B6* metabolizer phenotypes.

	Fast metabolizer mothers	Intermediate metabolizer mothers	Slow metabolizer mothers
Maternal efavirenz concentration (ng/mL)	1075 (698-1389) n = 22	1453 (938-2131) n = 45	4317 (3348-6246) n = 13
Newborn efavirenz concentration (ng/mL)			
Pooled	786 (9577-1060) n = 22	1183 (922-1815) n = 45	3028 (1746-3563) n = 13
Fast metabolizer newborns	853 (648-1054) n = 14	1130 (937-1714) n = 11	2914 n = 1
Intermediate metabolizer newborns	690 (485-1233) n = 8	1253 (730-1982) n = 25	2202 (1974-5718) n = 3
Slow metabolizer newborns	-	1298 (1142-1455) n = 9	3214 (3028-3895) n = 9
